# Enhancing wellbeing among pre-service teachers through a mindfulness-based social and emotional learning curriculum: a quasi-experimental study in China

**DOI:** 10.3389/fpsyg.2025.1405676

**Published:** 2025-01-22

**Authors:** Yunpeng Wu, Liping Qin

**Affiliations:** School of Teacher Education, Dezhou University, Dezhou, China

**Keywords:** cultural adaptation, mindfulness, pre-service teacher, social and emotional learning, teacher education, wellbeing

## Abstract

**Background:**

Integrating mindfulness practices and Social and Emotional Learning (SEL) into teacher education can significantly enhance educators’ wellbeing, resilience, and effectiveness. However, the cultural specificity of such interventions, particularly in the Chinese educational context, requires careful adaptation and examination.

**Objective:**

This study aims to design, implement, and evaluate a culturally adapted MBSEL curriculum for Chinese pre-service teachers. It investigates the program’s effectiveness in improving mindfulness, self-compassion, and life satisfaction, contributing to the literature on integrating mindfulness and SEL in teacher education within a specific cultural setting.

**Methods:**

Employing a quasi-experimental design, the study involved a sample of 41 Chinese pre-service teachers divided into an experimental group (*n* = 22) and a control group (*n* = 19). The MBSEL program’s impact on participants’ mindfulness, self-compassion, and life satisfaction was assessed using pre- and post-intervention measures.

**Results:**

The results of repeated measures analyses of variance indicated significant improvements in mindfulness, self-compassion, and life satisfaction among participants in the experimental group compared to the control group. These outcomes underscore the effectiveness of the culturally adapted MBSEL program in enhancing key aspects of pre-service teachers’ wellbeing and professional readiness.

**Discussion:**

The study demonstrates that a culturally adapted MBSEL curriculum effectively enhances mindfulness, self-compassion, and life satisfaction among Chinese pre-service teachers. This emphasizes the necessity of cultural sensitivity in developing mindfulness and SEL interventions for teacher education. Moreover, the success of the MBSEL program suggests its potential for global integration, urging policymakers and educators to prioritize culturally tailored approaches to enhance teacher wellbeing and educational outcomes.

## 1 Introduction

Teachers’ mental health is vital, yet they face numerous challenges that impact wellbeing, self-efficacy, and burnout (Agyapong et al., 2022; Cavionia et al., 2023). In China, mental health issues are prevalent among educators, with 60% of preschool teachers in Southwest China reporting distress and 40% of university teachers experiencing post-pandemic anxiety, particularly women and those facing economic hardships (Yao et al., 2022; Fu et al., 2022; Yu et al., 2022). The integration of Mindfulness-Based Social and Emotional Learning (MBSEL) frameworks, grounded in CASEL’s SEL model, offers a promising approach to mitigate these challenges. CASEL’s framework identifies five core competencies—self-awareness, self-management, social awareness, relationship skills, and responsible decision-making—that are essential for educators’ emotional and social development.

While mindfulness practices are well-documented for their stress-reducing benefits, integrating them with CASEL-based SEL strategies can further improve emotional regulation, empathy, and interpersonal skills (Durlak et al., 2011). However, China’s unique cultural and educational context poses distinct challenges that remain underexplored in research on integrated MBSEL programs. This study addresses these gaps by developing and evaluating a culturally adapted MBSEL curriculum tailored to Chinese pre-service teachers. The curriculum integrates mindfulness practices with SEL competencies to address CASEL’s five dimensions while focusing on three key outcome constructs: mindfulness, self-compassion, and life satisfaction. These outcomes were selected for their relevance to pre-service teachers in high-pressure environments: mindfulness as a foundation for SEL, self-compassion for emotional resilience, and life satisfaction as an indicator of overall wellbeing. This research introduces a culturally sensitive MBSEL framework, providing empirical evidence on its effectiveness in non-Western educational settings. By examining the impacts on mindfulness, self-compassion, and life satisfaction, the study contributes to the discourse on educator wellbeing and offers insights into culturally responsive practices for pre-service teachers in China and other educational environments globally.

The paper is structured as follows: The Literature Review establishes CASEL as a foundational framework and explores the theoretical integration of mindfulness and SEL. The Method section details participant selection and program implementation. The Results section examines the program’s impacts on mindfulness, self-compassion, and life satisfaction. The Discussion interprets these findings within the broader research context and outlines future directions. Finally, the Conclusion summarizes the findings and their global implications.

### 1.1 Key indicators of teacher’s wellbeing

For educators and pre-service teachers, mindfulness, self-compassion, and life satisfaction are closely interconnected and foundational to navigating the stresses and challenges of the teaching profession. These constructs align with the competencies outlined in CASEL’s SEL framework, which supports holistic wellbeing and professional growth.

Mindfulness is fundamental to enhancing teacher wellbeing. Introducing mindfulness in teacher preparation programs helps pre-service teachers develop the skills needed to handle the demands of teaching, leading to enhanced resilience and mental wellbeing (Dewhirst and Goldman, 2018). Mindfulness practices, such as Mindfulness-Based Stress Reduction (MBSR), have been shown to significantly reduce stress, anxiety, and symptoms of depression among teachers and pre-service teachers. For example, a randomized controlled trial found improvements in mental health and reduced stress among student teachers participating in mindfulness programs (Juul et al., 2021a). Mindfulness also improves teaching practices, including better classroom organization and instructional support, which correlate with reductions in negative affect and stress (Hirshberg et al., 2020).

Self-compassion serves as a crucial buffer against burnout and stress, promoting resilience and emotional wellbeing (Eriksson et al., 2018). Within the SEL framework, self-compassion relates strongly to self-awareness and self-management, as it encourages individuals to recognize and regulate their emotions while fostering a nurturing inner dialogue. Teachers with higher self-compassion are better at managing stress and building resilience. Compassion-focused interventions have been shown to significantly reduce symptoms of stress and improve professional wellbeing (Matos et al., 2022). This is particularly relevant for pre-service teachers, who often face heightened self-expectations during their professional training (Qin et al., 2022).

Life satisfaction reflects the broader impact of SEL on wellbeing. It incorporates all five SEL competencies by integrating emotional regulation, positive interpersonal relationships, and responsible decision-making into a sense of fulfillment and purpose. High levels of life satisfaction mitigate the risk of burnout and enhance long-term career satisfaction, contributing to a more stable teaching workforce (Garner et al., 2018). Pre-service teachers with greater life satisfaction tend to develop stronger coping mechanisms and resilience, which are essential for managing the demands of teacher training and the transition to professional teaching (Cayupe et al., 2023). Together, these three constructs form the foundation for teacher wellbeing, highlighting their critical importance in the development of resilient and effective educators.

### 1.2 Mindfulness in educational settings

Mindfulness, defined as the practice of maintaining a moment-by-moment awareness of our thoughts, feelings, bodily sensations, and surrounding environment with openness and non-judgment, has been increasingly recognized for its significant benefits across various educational contexts (Napoli et al., 2005; Meiklejohn et al., 2012). This practice enhances cognitive and emotional wellbeing (Schonert-Reichl et al., 2015), offering a transformative tool for educators and students alike.

For educators, mindfulness training has proven to enhance self-efficacy, emotional resilience, and classroom management skills, leading to supportive learning environments and effective teacher-student relationships (Meiklejohn et al., 2012). Studies have documented mindfulness’s role in reducing teacher burnout and stress, significantly contributing to improved occupational health and job satisfaction (Lomas et al., 2017; Roeser et al., 2012). For example, a six-week mindfulness-based program significantly increased mindfulness and wellbeing in pre-service teachers, reducing stress and symptoms of depression (Hue and Lau, 2015). Similarly, research highlights that mindfulness interventions can enhance teachers’ emotional regulation, coping strategies, and empathy, thereby improving their ability to positively evaluate and manage student behaviors (Shapiro et al., 2016; Taylor et al., 2016). These outcomes not only bolster teacher wellbeing but also set a foundation for enriched student engagement and academic success. Moreover, programs tailored for teachers, such as those aimed at supporting high-risk student populations, demonstrate mindfulness’s potential to enhance both teacher and student mental health (Juul et al., 2021b).

A systematic review underscores mindfulness interventions’ capacity to mitigate stress, improve wellbeing, and enhance teaching performance in in-service teachers (Hwang et al., 2017). These findings establish mindfulness as an essential component of professional development programs, highlighting its role in promoting sustainable teaching practices and a positive classroom environment. Students benefit from mindfulness through improved attention, working memory, emotional regulation, and reduced anxiety, contributing to better academic and social skills (Napoli et al., 2005; Meiklejohn et al., 2012). In higher education, mindfulness has been linked to increased calmness and reduced anxiety, indicating its comprehensive benefits across educational levels (Schwind et al., 2017).

Despite these documented benefits, a pressing call is for more rigorous and culturally nuanced research, particularly in settings like China. Integrating mindfulness practices with SEL, grounded in CASEL’s five competencies, offers a structured and holistic approach to addressing these challenges. The Chinese educational system’s emphasis on academic achievement has bolstered its reputation for high-performing students, while also highlighting the need for practices that foster emotional resilience and mental well-being. The cultural context of China, shaped by diverse philosophical traditions such as Confucianism, Taoism, and Buddhism, provides a nuanced foundation for integrating mindfulness into education. These traditions emphasize values such as harmony, balance, and self-reflection, which align closely with the principles of mindfulness and support its potential applicability in fostering a well-rounded educational experience. As Donahue-Keegan et al. (2019) suggest, integrating SEL and culturally responsive practices into teacher education provides a promising framework for addressing diverse educational challenges.

### 1.3 Effects of mindfulness-based interventions on teacher’s wellbeing

Mindfulness practices and SEL strategies provide complementary pathways to enhance these vital aspects of educators’ lives. Mindfulness, emphasizing present-moment awareness without judgment, serves as a foundation for cultivating self-awareness, emotional regulation, and self-compassion (Ferrari et al., 2019). SEL complements mindfulness by further developing emotional and interpersonal skills, supporting educators in managing stress, improving decision-making, and fostering positive relationships with colleagues and students. Together, mindfulness and SEL create a robust framework for enhancing self-compassion and life satisfaction, particularly in high-pressure environments like teacher education.

Mindfulness-based interventions (MBIs) and SEL strategies effectively target these constructs, addressing the professional and personal challenges faced by pre-service teachers. MBIs have been shown to significantly improve mental health outcomes in university students, particularly by reducing symptoms of anxiety, depression, and stress, while enhancing mindfulness and wellbeing (González-Martín et al., 2023; Gong et al., 2023; Chen et al., 2023). Studies suggest that while the effects of MBIs are generally small to moderate, their benefits for distress reduction and mindfulness enhancement may last beyond 3 months (Dawson et al., 2019; Galante et al., 2017).

Self-compassion also benefits from MBIs, as these interventions reduce burnout and compassion fatigue while fostering emotional resilience (Wasson et al., 2020; Neff and Germer, 2013). Research further indicates that mindfulness fosters adaptive coping strategies, such as increased emotional flexibility and greater self-esteem, which are essential for maintaining life satisfaction in high-stress professions like teaching (Galante et al., 2017).

In terms of life satisfaction, mindfulness and SEL programs improve emotional regulation, resilience, and positive affect, contributing to a greater sense of contentment in professional and personal domains (Carvalho et al., 2017; Crain et al., 2017). Programs integrating mindfulness and SEL competencies also enhance educators’ overall wellbeing and job satisfaction by promoting self-esteem and better coping mechanisms (Li et al., 2022; Kong et al., 2014; Wang and Kong, 2020).

Despite their documented benefits, the application of mindfulness and SEL practices in Chinese pre-service teacher education remains underexplored. This study addresses this gap by focusing on mindfulness, self-compassion, and life satisfaction—constructs highly relevant to the cultural and professional challenges faced by Chinese pre-service teachers. By examining the effects of culturally adapted mindfulness and SEL practices, this research offers strategies to enhance resilience, satisfaction, and professional competence, while contributing to the broader discourse on culturally responsive educator training. These findings hold promise for improving teacher wellbeing and fostering supportive educational environments globally.

### 1.4 Integration of SEL in teacher education and its importance

The integration of Social and Emotional Learning into teacher education programs plays a pivotal role in enhancing educational outcomes by fostering key competencies such as emotional understanding, empathy, goal setting, positive relationship building, and responsible decision-making (Payton et al., 2000). These skills are crucial for both academic success and personal growth, highlighting the importance of SEL in the comprehensive development of pre-service teachers.

Systemic SEL strategies, exemplified by the RULER approach, enhance teaching quality and student academic performance by offering extensive training for educators, integrating SEL into curricula, and fostering community engagement (Brackett et al., 2019). These strategies have proven effective in reducing teacher stress and burnout, directly benefiting the educational environment and student learning outcomes.

The impact of teachers’ social–emotional competence on students’ success is well documented, with interventions aimed at improving teachers’ emotional skills leading to a nurturing and effective learning environment (Schonert-Reichl, 2019). For pre-service teachers, SEL training is crucial for developing essential professional skills and classroom efficacy. It not only boosts emotional competencies such as empathy and self-esteem but also enhances social skills including assertiveness and communication, thereby reducing professional anxiety and improving classroom management (Palomera et al., 2017; Sugishita and Dresser, 2019).

### 1.5 Theoretical underpinning linking mindfulness with SEL approaches

The fusion of Mindfulness-Based Practices (MBP) with Social and Emotional Learning draws on their collective focus on cultivating self-awareness, emotional regulation, and interpersonal skills to bolster personal and social competencies. This alignment bridges mindfulness techniques, emphasizing present-moment awareness and non-judgmental observation, with SEL’s core components like self-awareness, self-management, and empathy, fostering a seamless integration of the two.

Mindfulness sharpens self-awareness, allowing for an objective observation of one’s thoughts and emotions, thereby facilitating personal strength recognition and emotional trigger identification crucial for SEL. It further supports self-management by enabling effective emotion regulation and stress management, aligning with SEL’s objectives. In fostering social awareness, mindfulness practices enhance empathy and understanding of others, complementing SEL’s focus on developing strong, positive relationships through effective communication and active listening. Moreover, mindfulness enhances responsible decision-making, encouraging reflective thought and mindful consideration of actions, paralleling SEL’s goal of cultivating a thoughtful, conscientious mindset.

This theoretical synergy underscores the potential of integrating MBP within SEL frameworks to not only enhance resilience and student wellbeing but also to educate the “whole child.” SEL’s emphasis on cognitive and emotional skills, combined with MBP’s focus on mindful awareness, proposes a comprehensive approach to education that nurtures positive self-concepts, moral values, and social–emotional understanding across K-12 settings (Gueldner and Feuerborn, 2016; Lawlor, 2016).

The integration of mindfulness and SEL offers a comprehensive framework to support personal and social growth. By combining mindfulness’s emphasis on awareness and regulation with SEL’s focus on skills like empathy and decision-making, this approach prepares individuals for academic success and life challenges. Together, they create a foundation for developing well-rounded, resilient learners with strong interpersonal and emotional competencies.

### 1.6 Enhancing educational outcomes with mindfulness-based SEL

The integration of Mindfulness-Based Social and Emotional Learning (MBSEL) offers a transformative approach to education, marrying mindfulness with social and emotional skills development to significantly benefit students and teachers alike. Mindfulness-based interventions (MBIs) are pivotal in promoting psychological wellbeing, self-regulation, positive emotional states, and stress reduction, showcasing their broad applicability and effectiveness in educational settings (Zhang et al., 2021; Brown and Ryan, 2003). This robust evidence base underscores the value of integrating mindfulness with SEL, highlighting an urgent need for further exploration across diverse conditions and populations.

Research supports the efficacy of integrating mindfulness into SEL, revealing that such training enhances attentional and emotional self-regulation, increases cognitive flexibility, and improves essential skills including working memory, academic performance, and emotional wellbeing (Meiklejohn et al., 2012). Additionally, mindfulness practices offer effective emotion regulation strategies, facilitating improved distress management and goal-directed behaviors (Roemer et al., 2015), which are critical components of successful SEL programs.

For teachers, mindfulness-based SEL programs advance emotional resilience, self-compassion, and overall professional wellbeing. Teachers who engage in these practices report greater self-kindness, reduced stress, and improved classroom management (Carvalho et al., 2017). SEL programs enriched with mindfulness have also been linked to enhanced cognitive control, reduced teacher burnout, and improved teacher-student relationships (Gueldner and Feuerborn, 2016; Schonert-Reichl et al., 2015). These benefits extend to pre-service teachers, equipping them with the emotional and cognitive tools needed to navigate professional challenges effectively (Garner et al., 2018).

Incorporating mindfulness into SEL frameworks offers a holistic strategy to enhance the wellbeing and effectiveness of educators and students alike. Evidence-based interventions in mindfulness and SEL not only alleviate stress but also develop key competencies such as self-awareness and empathy, crucial for fostering positive classroom dynamics and teacher-student relationships. By integrating these practices into teacher education, pre-service educators are better prepared to manage emotional demands, create supportive learning environments, and sustain professional resilience. This dual focus on emotional and professional growth underscores the transformative potential of MBSEL to elevate teaching quality and student outcomes in a sustainable manner, reaffirming its critical role in modern education.

### 1.7 The current study

Pre-service teacher education in China is shaped by a long-standing tradition which emphasize discipline, academic excellence, and respect for authority. While these values contribute to a strong professional foundation, pre-service teachers often face considerable challenges, including demanding academic requirements and a substantial workload. These factors can lead to stress and burnout, posing risks to their overall wellbeing. Additionally, existing teacher training programs often lack comprehensive exposure to Social and Emotional Learning (SEL), which is critical for fostering effective emotion management and positive student engagement. Research indicates that while pre-service teachers often begin their training with high levels of motivation, prolonged exposure to these stressors may diminish their enthusiasm over time (Ye et al., 2021).

This research aims to develop and evaluate a Mindfulness-Based Social and Emotional Learning (SEL) curriculum tailored for pre-service teachers, addressing the need for culturally responsive education practices. While the study has a particular focus on China, its findings aim to offer insights applicable to diverse educational contexts, emphasizing the importance of cultural sensitivity and the multifaceted impacts of mindfulness-based interventions on wellbeing.

Central to our research are two primary objectives: first, to adapt mindfulness-based SEL practices to align more closely with the cultural and educational realities of Chinese pre-service teachers, thereby increasing the curriculum’s relevance and effectiveness for this group. This goal acknowledges the predominance of Western-centric literature in this field and highlights the significance of cultural adaptability in SEL practices globally. Second, the study explores how mindfulness and SEL affect self-compassion and life satisfaction among pre-service teachers. To systematically investigate these effects, we propose the following hypothesis:

Hypothesis 1 (*H1*): Pre-service teachers participating in the culturally adapted MBSEL curriculum will exhibit significant improvements in mindfulness compared to a control group.

Hypothesis 2 (*H2*): Pre-service teachers undergoing the MBSEL curriculum will significantly increase self-compassion post-intervention.

Hypothesis 3 (*H3*): Engagement in the MBSEL program will significantly enhance life satisfaction among participants in the experimental group.

Our study utilizes a quasi-experimental design to explore the interconnected outcomes of mindfulness and Social and Emotional Learning (SEL), specifically targeting strategies that enhance wellbeing in educational settings globally. We have developed a curriculum tailored to the cultural and educational needs of Chinese pre-service teachers, addressing a significant gap in the literature and offering insights into international educational practices. By focusing on these objectives, our research aims to enrich the body of knowledge and provide practical solutions for enhancing the wellbeing, resilience, and professional development of educators worldwide. Through this work, we aspire to contribute significant theoretical and practical advancements to the field of teacher education.

## 2 Materials and methods

### 2.1 Participants

We employed a random cluster sampling method for this study. Participants were students enrolled in a Bachelor of Teacher Education program at a university in North China, where the term ‘Shīfàn sheng’ refers to preservice teachers. These students typically enroll in university programs that prepare them for careers as professional educators. For this study, intervention participants were those who elected to enroll in a course focused on Mindfulness-Based Social and Emotional Learning (MBSEL), while control participants, matched in majors and academic levels, did not enroll in this specific course or any other similar program.

Participants included in the study were required to be enrolled in a pre-service teacher training program, be between 18 and 22 years old, and provide informed consent to participate. Participants were excluded if they had prior extensive training in mindfulness or psychological counseling, to avoid confounding effects of previous similar interventions. Additionally, participants with severe psychological conditions requiring clinical intervention, as determined by a preliminary screening questionnaire, were also excluded.

To determine the appropriate sample size for our study, we conducted a preliminary power analysis using G*Power 3.1. The analysis was based on the following parameters: an expected medium effect size (*f* = 0.25), an alpha level of 0.05, a desired power of 0.80, two groups (experimental and control), and two measurement points (pre-test and post-test). We assumed a correlation among repeated measures of 0.5 and a nonsphericity correction epsilon (*ε*) of 1, indicating perfect sphericity. The G*Power analysis indicated that a total sample size of 34 participants would be required to achieve the desired power. This translates to 17 participants per group. The actual power achieved with this sample size is 0.80, ensuring that the study is adequately powered to detect a medium effect size.

Our final sample consisted of 41 participants, with 22 in the intervention group (95% female) and 19 in the control group (95% female). Given that our target population predominantly comprises female students, reflecting the demographic characteristics of education majors, the sample is highly representative of the population. The mean (SD) age was 19.7 (0.7) for the intervention group and 19.4 (0.6) for the control group. No significant demographic differences were observed between the two groups, suggesting that the results could be generalized across similar demographics within the population studied.

### 2.2 Procedures

Before commencement, the study received approval from the Ethics Committee of the author’s University, adhering to the principles outlined in the Declaration of Helsinki. Participation in the study was voluntary, and all students provided informed consent by signing consent forms.

Data collection involved administering a battery of questionnaires to participants both before and immediately after the conclusion of the course. Questionnaires were administered to students in group settings, within the school premises, during pre-arranged class periods. The researcher assured participants that their responses would be kept anonymous and confidential, fostering an environment conducive to open and honest feedback.

### 2.3 MBSEL curriculum design

Drawing on the framework Lawlor (2016) outlined, the program was designed to deepen competencies such as Self-awareness, Self-management, Social awareness, Relationship skills, and Responsible decision-making. Lawlor’s framework bridges mindfulness practices and SEL competencies, providing a theoretical foundation that aligns mindfulness with the five core SEL dimensions identified by CASEL. By incorporating mindfulness practices such as breathing exercises, movement-based techniques, and reflective activities, this framework enhances emotional regulation, empathy, and decision-making. Empirical studies support the applicability of Lawlor’s framework in diverse contexts. For instance, Feuerborn and Gueldner (2019) confirmed the conceptual fit between mindfulness-based practices (MBPs) and SEL competencies, particularly in areas like self-management and self-awareness. Gueldner and Feuerborn (2016) further demonstrated the practical integration of MBPs into established SEL curricula, such as Strong Kids and Strong Teens, highlighting its flexibility and effectiveness in fostering resilience and wellbeing.

To address the unique needs of pre-service teachers, the MBSEL program builds on evidence from previous research demonstrating the efficacy of mindfulness-based interventions (MBIs) in improving teacher wellbeing and professional readiness. For example, Hue and Lau (2015) implemented a six-week mindfulness-based program for pre-service teachers, which resulted in significant improvements in mindfulness, stress reduction, and reduced symptoms of depression. Similarly, Hwang et al. (2017) highlighted how MBIs can enhance emotional regulation, coping strategies, and empathy, which are critical for managing classroom demands. The CARE program by Jennings et al. (2017) demonstrated that integrating mindfulness into teacher training can cultivate awareness and resilience, improving classroom management and teacher-student interactions. These studies collectively underscore the importance of equipping pre-service teachers with tools to manage stress and foster resilience, which served as a foundational principle for the MBSEL design.

To ensure the curriculum was culturally relevant and contextually appropriate, activities were carefully selected based on interviews with Chinese university students. The curriculum placed particular emphasis on Self-awareness, Self-management, and Relationship skills, as these areas were identified as critical for addressing the unique challenges faced by pre-service teachers. This design formed the foundation for an eight-week elective course that integrates mindfulness practices into the development of SEL competencies.

#### 2.3.1 Connecting mindfulness practices to SEL constructs

The MBSEL curriculum systematically aligns mindfulness practices with the five components of Social and Emotional Learning (SEL) identified by CASEL, ensuring each activity explicitly supports one or more competencies. This structured framework maps each practice to SEL dimensions, fostering emotional, social, and cognitive development (Table 1). Each activity is thoughtfully selected and sequenced to align with weekly objectives and broader program goals, ensuring coherence and systematic integration. For more detailed information on the activities, please refer to the Appendix.

**Table 1:** Design of the mindfulness-based social and emotional learning curriculum.

SEL competency	Mindful awareness	Week	Mindfulness practices 1	Mindfulness practices 2
Self-awareness	Understanding the nature of mind; Emotional awareness	1	Focused Mindful Breathing	Mindful Walking
		2	Body Scan Meditation	Zhan Zhuang (standing meditation)
Self-management	Emotion regulation; Inhibitory control; Deployment of attention	3	Square Breathing (Box breathing)	Chinese Ba-Duan-Jin practice (Eight Pieces of Brocade)
		4	Tai Chi	Red-Green Light Game
Social awareness	Showing empathy and compassion for others	5	Compassion/loving-kindness meditation	Empty Chair Practice
Relationship skills	Meaningful and reciprocal dialogue; Managing conflict	6	Mindful Listening and Communication Practice	Social Role-Playing
		7	Emotions-observing Meditation	Mindful Communication Journal
Responsible decision-making	Stating facts without judgment; Making ethical choices based in awareness and caring	8	Five Senses Treasure Hunt	Active witnessing

Adapted from Lawlor (2016, p. 69).

Self-awareness: Activities such as Mindful Breathing, Body Scan Meditation, Mindful Walking, and Zhan Zhuang (standing meditation) help students develop self-awareness by fostering a non-judgmental observation of their internal experiences and bodily sensations. Mindful Breathing focuses on the natural rhythm of the breath, cultivating present-moment awareness and attentiveness. Body Scan Meditation encourages students to systematically observe physical sensations, building a stronger connection between mind and body. Mindful Walking introduces purposeful movement to sharpen focus and integrate mindfulness into daily activities. Zhan Zhuang (standing meditation) emphasizes grounding and posture, helping students notice subtle bodily sensations while cultivating calmness and balance. These practices collectively enhance students’ ability to recognize their emotions, thoughts, and physical states, aligning with CASEL’s focus on self-awareness as a foundation for SEL.

Self-management: For self-management, activities such as Box Breathing, Tai Chi, Ba-Duan-Jin (Eight Pieces of Brocade), and the Red-Green Light Game provide students with tools for emotional regulation and attention control. Box Breathing uses rhythmic breath control to calm the nervous system and improve focus. Tai Chi and Ba-Duan-Jin integrate mindful movement with controlled breathing to reduce stress and improve balance. The Red-Green Light Game enhances inhibitory control by requiring students to pause and reflect before acting, fostering thoughtful responses to stimuli. These practices align directly with SEL competencies related to managing emotions, controlling impulses, and maintaining focus under stress.

Social awareness: Practices such as Compassion/Loving-Kindness Meditation and the Empty Chair Practice enhance students’ social awareness by fostering empathy and perspective-taking. Compassion/Loving-Kindness Meditation guides students to generate goodwill toward themselves and others, cultivating emotional connections and a sense of shared humanity. The Empty Chair Practice allows students to imagine interactions from another person’s perspective, deepening their understanding of others’ emotions and viewpoints. These practices strengthen students’ ability to navigate social dynamics with sensitivity and care, aligning with CASEL’s emphasis on social awareness.

Relationship skills: Activities such as Mindful Listening and Communication Practice, Social Role-Playing, Emotions-Observing Meditation, and the Mindful Communication Journal support the development of meaningful and reciprocal relationships. Mindful Listening provides a structured approach to focusing entirely on another person’s words, fostering trust and mutual understanding. Social Role-Playing allows students to rehearse effective responses to interpersonal challenges in a safe and supportive environment. Emotions-Observing Meditation trains students to recognize and regulate emotions during interactions, and the Mindful Communication Journal enables reflective practice on communication experiences, helping students identify areas for growth. Together, these activities promote the SEL competency of building healthy and collaborative relationships.

Responsible decision-making: Activities such as the Five Senses Treasure Hunt and Active Witnessing help students develop skills for making thoughtful and ethical decisions. The Five Senses Treasure Hunt trains students to observe their environment using sensory awareness, fostering attention to detail and non-judgmental observation. Active Witnessing involves listening and observing with empathy, helping students reflect on the ethical and social implications of their actions. These practices enhance students’ ability to weigh consequences and align their choices with personal and social values, supporting CASEL’s focus on responsible decision-making.

#### 2.3.2 Cultural responsiveness in design

By integrating traditional practices and tailoring its content to the specific cultural context of Chinese university students, the MBSEL curriculum exemplifies a strong commitment to cultural responsiveness. A key feature of this approach is the integration of traditional Chinese mindfulness techniques, such as Zhan Zhuang (standing meditation) and Ba-Duan-Jin (Eight Pieces of Brocade), which resonate deeply with the cultural heritage of Chinese students. These practices provide familiar and meaningful entry points into mindfulness, enabling students to engage with activities that reflect their cultural identity and lived experiences. This alignment fosters a sense of comfort, belonging, and connection, enhancing both engagement and the overall learning experience. The goal was not to master these traditional disciplines but to use their structured movements as a vehicle for mindfulness and SEL development.

To enrich the mindfulness practices in the MBSEL curriculum, traditional Chinese exercises such as Ba Duan Jin and Tai Chi were incorporated. These practices were introduced not as comprehensive training programs but as accessible exercises that align with the broader objectives of Social and Emotional Learning (SEL), particularly in cultivating self-awareness and self-management. The primary purpose of incorporating traditional exercises like Ba Duan Jin and Tai Chi is not to assess their physiological impacts or delve deeply into their rich philosophical underpinnings. Instead, these practices serve as structured mindfulness activities, leveraging their rhythmic movements and controlled breathing to enhance participants’ self-awareness, emotional regulation, and overall mindfulness. The focus remains on SEL competencies rather than mastering the deeper intricacies of these traditional practices.

During classroom sessions, instructional videos and guided meditation audios from renowned practitioners were utilized as references, ensuring the accuracy and authenticity of the movements and enhancing the mindfulness experience. Facilitators, while not certified experts in these disciplines, had sufficient practice and familiarity with the exercises to guide participants effectively. For example, they demonstrated the movements based on established models and provided basic instructions on posture, breathing, and mindfulness integration. Participants were not required to achieve proficiency in these exercises. Instead, the emphasis was on using these activities as experiential tools to connect with SEL competencies, particularly self-awareness.

The integration of other traditional practices, such as Mindful Walking and Zhan Zhuang (standing meditation), followed a similar rationale, emphasizing their role as accessible and culturally resonant tools within the mindfulness-based SEL framework. These activities were chosen for their accessibility and alignment with the goals of mindfulness-based SEL, rather than as standalone disciplines requiring advanced expertise or long-term mastery. The curriculum leverages their form and simplicity to foster present-moment awareness and emotional regulation. By incorporating traditional Chinese exercises as supplementary tools, the MBSEL curriculum maintains a balance between cultural relevance and practical feasibility. This approach ensures that participants can engage meaningfully with these practices without necessitating advanced training or extensive time commitments.

The curriculum further adapts its content to reflect the values and lived experiences of Chinese students. For instance, role-play scenarios and ethical decision-making exercises incorporate culturally significant themes, including respect for authority, family obligations, and collective responsibility. These themes align with SEL competencies such as social awareness and responsible decision-making, ensuring that mindfulness practices are both theoretically grounded and practically relevant to students’ lives. By linking these exercises to culturally relevant contexts, the program encourages students to view mindfulness as a tool for addressing real-world challenges.

In addition, the curriculum reflects traditional Chinese philosophies, such as Confucian ideals of harmony and balance. These philosophies are included in discussions and teaching materials, creating a clear connection between cultural values and SEL principles. For example, the concept of harmony aligns with CASEL’s focus on relationship skills, fostering meaningful connections and conflict resolution in a culturally informed way. Language also plays a crucial role in improving accessibility and engagement. The program incorporates idioms, metaphors, and examples drawn from Chinese culture, allowing students to better understand and connect with the content on a personal level.

#### 2.3.3 Weekly class structure

The course was structured with two 45-min sessions per week, providing a consistent framework for learning and practice. The first session began with a 10-min introduction to SEL theory, providing students with a conceptual understanding of mindfulness practices. This was followed by a 15-min review of the previous week’s practice, during which the students exchanged their experiences and asked questions. The remaining 20 min were dedicated to introducing and practicing the first mindfulness activity of the week. The second session began with the introduction and practice of a second mindfulness activity that lasted 20 min. The session ended with a 25-min integrated exercise in which students combined the two activities sequentially. This structure reinforced the week’s learning and allowed students to deepen their understanding of the practices. Students were encouraged to complete at least one of the two weekly activities independently and log their reflections online. Partner-based activities in Weeks 6 and 8 added a collaborative element that focused on relationship skills and provided opportunities for peer learning.

#### 2.3.4 Curriculum iteration and feedback

The MBSEL curriculum has been continuously improved based on feedback from pre-service teachers. At the beginning of the program, focus group participants suggested incorporating traditional Chinese practices such as Tai Chi and Zhan Zhuang. These additions increased the program’s cultural relevance and strengthened its connection to students’ heritage. Midway through the course, students provided feedback on ways to improve classroom interaction and access to digital resources such as practice audio. These suggestions were implemented and resulted in significant improvements to the learning experience. Surveys conducted before and after these changes showed an increase in student satisfaction, with scores rising from 8.2 to 9.4. These adjustments not only made the program more engaging, but also enriched the classroom atmosphere and created a more effective and enjoyable learning environment. Through these iterations, the MBSEL curriculum has become a well-rounded program that effectively combines mindfulness practices with SEL principles while remaining culturally sensitive and responsive.

### 2.4 Outcome measures

#### 2.4.1 Mindfulness

The Chinese version of the Five Facet Mindfulness Questionnaire (FFMQ-C) has been validated and shown to have acceptable psychometric properties, making it a reliable instrument for assessing mindfulness among Chinese individuals. This questionnaire consists of five facets that measure different aspects of mindfulness: observing, describing, acting with awareness, nonjudging of inner experience, and nonreactivity to inner experience (Zhu et al., 2023). Items were rated on a 5-point rating scale ranging from 1 (never or very rarely true) to 5 (very often or always true). For the present study, Cronbach’s alphas at pre-test and post-test for the observing subscale were 0.84 and 0.83 respectively, for the describing subscale 0.83 and 0.85, respectively, for the acting with awareness subscale 0.84 and 0.86, respectively, for the nonjudging of inner experience subscale 0.77 and 0.82, respectively, and for the nonreactivity to inner experience subscale 0.82 and 0.79, respectively.

#### 2.4.2 Self-compassion

The Chinese version of The Self-Compassion Scale (SCS-C) has been validated and shown to have good psychometric properties, making it a reliable tool for assessing self-compassion among Chinese individuals. This scale measure three aspects of self-compassion, including self-kindness, common humanity, and mindfulness (Zhao et al., 2023). The participants rated their personal feelings and attitudes on a 5-point frequency scale, ranging from 1 (never) to 5 (always). For the present study, Cronbach’s alphas at pre-test and post-test for the self-kindness subscale were 0.77 and 0.78 respectively, for the common humanity subscale 0.82 and 0.80, respectively, and for the mindfulness subscale 0.85 and 0.87, respectively.

#### 2.4.3 Satisfaction with life

The Chinese version of the Satisfaction with Life Scale (SWLS-C) has been developed and validated as a reliable instrument for assessing life satisfaction among Chinese individuals (Bai et al., 2011). Participants rated their responses on a 7-point rating scale ranging from 1 (strongly disagree) to 7 (strongly agree). Cronbach’s alphas at the pre-test and post-test for the scale were 0.84 and 0.83, respectively.

## 3 Results

Before conducting primary analyses, we checked for normality assumptions by calculating skewness and kurtosis for all study variables. All variables met the criteria for normality (skewness and kurtosis within ±2). The Means, standard deviations, and correlations of dependent variables for all participants at pre-test and post-test periods were listed in Table 2.

**Table 2:** Means (M), standard deviations (SD), and normality tests of the study variables (Skewness and Kurtosis) for all participants at pre-test and post-test periods.

Variable	1	2	3	4	5	6	7	8	9
1. FFMQ-C obs 1	1								
2. FFMQ-C des 1	0.148	1							
3. FFMQ-C awa 1	−0.094	0.526	1						
4. FFMQ-C nonjud 1	0.201	0.284	0.404	1					
5. FFMQ-C non-rea 1	0.172	0.312	0.477	0.538	1				
6. SCS-C self-kind 1	−0.117	0.282	0.277	0.333	0.222	1			
7. SCS-C com hum 1	−0.071	0.263	0.404	0.222	0.351	0.392	1		
8. SCS-C mind 1	0.342	0.095	0.135	0.179	0.129	0.46	0.174	1	
9. SWLS-C 1	0.149	0.102	0.126	0.196	0.236	0.221	0.438	0.421	1
Mean	24.73	24.66	25.73	22.78	24.56	10.12	11.05	17.37	21.27
SD	5.56	5.08	6	4.49	5.27	2.45	3.81	3.33	5.1
Skewness	−0.08	−0.46	−0.07	−0.14	−0.28	−0.3	0.22	−0.17	−0.61
Kurtosis	0.28	0.58	−0.25	−0.17	−0.51	−0.14	−0.18	−0.72	0.37

Variable	10	11	12	13	14	15	16	17	18
10. FFMQ-C obs 2	1								
11. FFMQ-C des 2	0.203	1							
12. FFMQ-C awa 2	0.071	0.442	1						
13. FFMQ-C nonjud 2	0.245	0.453	0.513	1					
14. FFMQ-C non-rea 2	0.133	0.364	0.43	0.549	1				
15. SCS-C self-kind 2	−0.004	0.399	0.359	0.377	0.278	1			
16. SCS-C com hum 2	−0.323	0.093	0.528	0.278	0.342	0.507	1		
17. SCS-C mind 2	0.213	0.202	0.211	0.215	0.207	0.556	0.098	1	
18. SWLS-C 2	0.088	0.17	0.373	0.231	0.303	0.489	0.305	0.62	1
Mean	26.83	25.93	26.95	23.8	25.9	11.17	11.32	19.22	23.15
SD	4.95	4.96	5.11	4.37	5.34	2.27	3.89	3.42	5.48
Skewness	0	−0.07	0.3	−0.15	−0.24	−0.54	0.33	−0.44	−0.21
Kurtosis	−0.22	−0.46	−0.4	−0.07	−0.46	0.94	−0.36	−0.39	−0.38

FFMQ-C, The Chinese version of the Five Facet Mindfulness Questionnaire; obs, observing; des, describing; awa, acting with awareness; nonjud, nonjudging of inner experience; non-rea, non-reactivity to inner experience; SCS-C, The Chinese version of The Self-Compassion Scale; self-kind, self-kindness; com hum, common humanity; mind, mindfulness; SWLS-C, The Chinese version of the Satisfaction With Life Scale; 1 = pretest, 2 = post-test; SD, standard deviations.

We conducted independent sample t-tests to explore the differences between groups on each outcome measure before the training commenced. Our analysis revealed a significant pre-training difference between groups in the non-reactivity to inner experience facet of mindfulness (*t* = −3.549, *p* = 0.001) and satisfaction with life (*t* = −2.106, *p* = 0.042). These results suggest that the experimental group exhibited lower scores compared to the control group for both of these outcome variables.

To examine intervention effectiveness, we performed 2 (groups: experimental, control) by 2 (pre vs. post intervention) repeated measures (RM) analyses of variance (ANOVAs) with partial eta squared (η_p_^2^) as an indicator of effect size for group by time interactions, where values of 0.01, 0.06, and 0.14 are associated with small, medium, and large effects, respectively (Olejnik and Algina, 2000). We chose to use RM ANOVA (Repeated Measures Analysis of Variance) over RM MANOVA (Repeated Measures Multivariate Analysis of Variance) for several reasons. Firstly, our primary interest was to examine the effectiveness of the intervention on individual outcome measures rather than their combined effect. RM ANOVA allows us to focus on the differences in means for each dependent variable across time and groups, aligning with our specific hypotheses. Secondly, RM ANOVA provides straightforward results that are easier to interpret and communicate, particularly for the individual effects of each dependent variable. This clarity is beneficial for applied research contexts where understanding specific variable changes is crucial. Thirdly, while RM MANOVA is robust against violations of sphericity and accounts for the intercorrelations among dependent variables, it requires larger sample sizes and more complex interpretation. Given our sample size and research objectives, RM ANOVA was more appropriate. Lastly, although RM MANOVA could be used to account for the interdependencies among multiple outcome measures, it would add complexity without significantly enhancing the interpretation of our results. The focus of our study is on the individual changes in mindfulness, self-compassion, and satisfaction, which RM ANOVA adequately addresses.

In addition to normality, assumptions for using RM ANOVA, including sphericity, and homogeneity of variance, were tested using the Mauchly’s test of sphericity and Levene’s test, respectively, and were found to be satisfactory. As shown in Table 3, the RM ANOVA analysis indicated the intervention had the hypothesized influence on mindfulness (observing, acting with awareness, non-judging, and non-reactivity to inner experience dimensions), self-compassion (self-kindness and mindfulness dimensions), and satisfaction with life as indicated by a significant group by time interaction favoring experimental students. The effect sizes for these interactions were medium to large.

**Table 3:** Means scores and indicators of intervention effectiveness for the Mindfulness-Based SEL curriculum.

Outcome measure	Pretest		Posttest		Group and time interaction indicators		
	*M*	SD	*M*	SD	*F*	*p*	η_p_^2^
FFMQ-C observing					12.22	0.001	0.239
Experimental (*n* = 22)	23.41	4.58	27.68	5.13			
Control (*n* = 19)	26.26	6.29	25.84	4.68			
FFMQ-C describing					1.18	0.284	0.029
Experimental (*n* = 22)	25.59	4.28	27.55	4.74			
Control (*n* = 19)	23.58	5.81	24.05	4.65			
FFMQ-C acting with awareness					5.95	0.019	0.132
Experimental (*n* = 22)	25.68	6.66	28.41	5.02			
Control (*n* = 19)	25.79	5.33	25.26	4.79			
FFMQ-C non-judging of inner experience					5.86	0.020	0.131
Experimental (*n* = 22)	21.77	5.42	24.36	4.50			
Control (*n* = 19)	21.74	3.33	22.05	4.21			
FFMQ-C non-reactivity to inner experience					5.57	0.023	0.125
Experimental (*n* = 22)	18.41	3.19	21.82	4.15			
Control (*n* = 19)	21.63	2.52	22.58	2.71			
SCS-C self-kindness					4.27	0.045	0.099
Experimental (*n* = 22)	9.91	2.74	11.68	2.50			
Control (*n* = 19)	10.37	2.11	10.58	1.87			
SCS-C common humanity					2.11	0.154	0.051
Experimental (*n* = 22)	10.73	4.322	11.64	4.686			
Control (*n* = 19)	11.42	3.203	10.95	2.778			
SCS-C mindfulness					10.61	0.002	0.214
Experimental (*n* = 22)	16.50	3.189	19.45	3.863			
Control (*n* = 19)	18.37	3.287	18.95	2.896			
SWLS-C					10.26	0.003	0.208
Experimental (*n* = 22)	19.77	5.55	23.23	5.960			
Control (*n* = 19)	23.00	4.00	23.05	5.027			

FFMQ-C, The Chinese version of the Five Facet Mindfulness Questionnaire; SCS-C, The Chinese version of The Self-Compassion Scale; SWLS-C, The Chinese version of the Satisfaction With Life Scale.

To better analyze the impact of the intervention, we performed simple effects test for Time. Results indicated that in the experimental group, the simple effects for Time was significant for observing, *F* = 21.85, *p* < 0.001, partial η^2^ = 0.359, acting with awareness, *F* = 9.03, *p* = 0.005, partial η^2^ = 0.188; non-judging, *F* = 16.39, *p* = 0.000, partial η^2^ = 0.296, non-reactivity to inner experience, *F* = 23.07, *p* < 0.001, partial η^2^ = 0.372; self-kindness, *F* = 11.88, *p* = 0.001, partial η2 = 0.233, mindfulness, *F* = 35.40, p = 0.000, partial η^2^ = 0.476; SWL, *F* = 22.84, *p* = 0.000, partial η^2^ = 0.369, with the post-test scores being significantly higher than the pre-test scores. In the control group, the simple effects of Time for those outcome variables were not significant.

In sum, the results of this study provide robust support for all three hypotheses, demonstrating the effectiveness of the MBSEL intervention in enhancing mindfulness, self-compassion, and life satisfaction among pre-service teachers. Significant improvements were observed across the four dimensions of mindfulness—observing, acting with awareness, non-judging, and non-reactivity to inner experience—with medium to large effect sizes in the experimental group. Similarly, self-compassion showed significant gains in the dimensions of self-kindness and mindfulness, indicating the program’s role in fostering emotional resilience. Life satisfaction also significantly improved in the experimental group, highlighting the intervention’s broad impact on overall wellbeing. Notably, pre-test comparisons revealed that the experimental group initially scored lower in non-reactivity to inner experience and life satisfaction compared to the control group, underscoring the program’s effectiveness in addressing these deficits. In contrast, no significant changes were observed in the control group across these measures.

## 4 Discussion

Our study’s exploration of a Mindfulness-Based Social and Emotional Learning (MBSEL) program tailored for Chinese pre-service teachers has yielded notable improvements across several key areas. Specifically, the program influenced four out of five measured dimensions of mindfulness, including observing, acting with awareness, non-judging, and non-reactivity to inner experience. In terms of self-compassion, significant gains were observed in two out of the three dimensions assessed, namely self-kindness and mindfulness. Additionally, participants in the experimental group showed improvements in their overall life satisfaction, demonstrating the holistic benefits of the intervention.

Our hypotheses were tested with a series of analyses, all proposed hypotheses were supported. These findings align with and extend the body of literature that underscores the benefits of integrating mindfulness and SEL practices within educational settings (Meiklejohn et al., 2012; Lawlor, 2016). The observed effect sizes ranged from moderate to large, suggesting that the MBSEL program significantly enhanced the measured outcomes. However, these effect sizes were slightly smaller than those reported in some previous studies (Carvalho et al., 2017; Roemer et al., 2015), likely due to the relatively small sample size of our study. While the results clearly demonstrate the program’s substantial impact on pre-service teachers’ wellbeing and professional readiness, the limited sample size may have constrained the magnitude of the observed effects.

These improvements are particularly significant for pre-service teachers, whose future roles in educational settings demand emotional resilience and mindfulness, increasingly recognized as foundational competencies for effective teaching. Even within the constraints of a smaller sample size, the moderate to large effects observed underscore the practical relevance of integrating mindfulness and SEL into teacher education, emphasizing its potential to support the personal and professional growth of pre-service teachers in dynamic and demanding environments.

First, the observed enhancements in mindfulness, particularly in the non-reactivity to inner experience facet, supported our first hypothesis (H1). This supports prior research suggesting that mindfulness training fosters an individual’s ability to experience thoughts and emotions without immediate, reactive responses (Kabat-Zinn, 1994). This capability is invaluable for educators who often face stressful situations requiring composed and thoughtful reactions. Our findings indicate that participants were able to cultivate a more observant and less reactive stance toward their experiences, an essential skill for navigating the challenges of teaching (Roeser et al., 2012).

Second, the significant gains in self-compassion supported our second hypothesis (H2) and highlighted the effectiveness of the MBSEL program in fostering emotional resilience among pre-service teachers. Excessive self-demands, such as perfectionism, can lead to academic stress and burnout among pre-service teachers (Qin et al., 2022). Meanwhile, reducing stress can enhance an individual’s academic performance and wellbeing (Wijaya et al., 2022). Considering the educational environment in China, which often emphasizes perfection and high performance, fostering self-compassion can provide a buffer against stress and burnout (Ferrari et al., 2019). This finding supports the notion that self-compassion training can be an integral component of teacher education, promoting emotional resilience.

Third, the increase in life satisfaction among participants supports our third hypothesis (H3), indicating the holistic benefits of integrating mindfulness and SEL into teacher training. Life satisfaction, as a broad measure of wellbeing, reflects the participants’ improved perception of their quality of life and overall happiness. This improvement is particularly noteworthy given the highly professional and rigorous nature of the teaching profession in China. It suggests that mindfulness and SEL skills not only enhance specific competencies but also contribute to a more satisfying and fulfilling personal and professional life (Kong et al., 2014).

Improvements in mindfulness, self-compassion, and life satisfaction underscore the critical role of mindfulness and SEL in managing stress and mitigating educator burnout, aligning with findings by Jennings and Min (2023). These outcomes also illustrate the relevance of Lazarus and Folkman’s Stress and Coping Model within educational contexts, which emphasizes using both problem-focused strategies to address stressors and emotion-focused strategies to regulate emotional responses (Folkman, 2013). Mindfulness and SEL practices empower educators to adopt this balanced approach, enhancing their ability to manage stress effectively. Notably, the non-reactivity to inner experience facet of mindfulness highlights its value in helping educators maintain composure in challenging teaching environments.

While the control group did not exhibit significant changes, the experimental group’s positive outcomes underscore the MBSEL program’s effectiveness. This study demonstrates the value of culturally adapted mindfulness-based SEL interventions in enhancing educators’ wellbeing and professional competence. The findings suggest a need to integrate evidence-based mindfulness and SEL strategies more broadly, with the potential to transform teacher training practices not just in China but globally. Such interventions can provide a universally applicable framework for fostering educator resilience, satisfaction, and competence in diverse educational contexts.

### 4.1 Practical implications for education

This study highlights the importance of cultural adaptation in the Mindfulness-Based Social and Emotional Learning (MBSEL) curriculum for Chinese pre-service teachers. The program draws from China’s cultural traditions, including Confucianism, Taoism, and Buddhism. Practices like “Zhan Zhuang” (standing meditation) and “Tai Chi” are incorporated to resonate with these traditions. MBSEL blends these approaches with established mindfulness and SEL techniques. By addressing local values and cultural contexts, the curriculum offers a tailored and responsive framework for teacher education in China.

Currently, systematic implementation of SEL programs in Chinese higher education teacher training remains rare. Most teacher education courses focus heavily on subject knowledge and teaching skills, with relatively little emphasis on emotional regulation and mental health support for teachers. The MBSEL program, by integrating mindfulness practices with core SEL competencies, addresses this gap and aligns with recent policy directions emphasizing the enhancement of teacher psychological wellbeing and emotional management skills. For example, policy documents from the Ministry of Education advocate for strengthening social–emotional competencies and resilience in teacher preparation programs. As NPC Representative Mei Bing suggested, SEL should be systematically incorporated into pre-service and in-service teacher training with additional SEL-related metrics included in professional and curricular standards (Cha and Chen, 2024). The MBSEL program reflects this policy direction by equipping pre-service teachers with theoretical knowledge and practical tools to support their wellbeing and that of their students.

To ensure effective integration into teacher education systems, MBSEL can be implemented flexibly as an elective course, an interdisciplinary training module, or a thematic workshop. These formats allow institutions to adapt the program to their specific needs, offering pre-service teachers opportunities to engage with SEL principles and practices in diverse contexts. Building on pilot experience, several practical recommendations can guide its implementation. Cultural adaptation remains critical; integrating region-specific examples, culturally familiar metaphors, and localized activities can enhance the program’s relevance. Comprehensive resource preparation is also essential, including training qualified facilitators and developing accessible teaching materials such as instructional videos and guided meditation audios. Collaboration with policymakers and partnerships between educational institutions, research centers, and schools will ensure alignment with standards and provide institutional support for sustainability.

Moreover, MBIs can be delivered in various formats, including traditional in-person sessions, online programs, and mobile mindfulness meditation (MMM) apps, which provide accessible options for university students and pre-service teachers alike (Gong et al., 2023; Yosep et al., 2024; Chen et al., 2023). While digital interventions like MMM apps may be less conclusive for depression outcomes, they have shown efficacy in reducing stress and anxiety, promoting mindfulness, and enhancing overall wellbeing (Yosep et al., 2024). These flexible delivery formats address the logistical and emotional challenges faced by pre-service teachers, enabling wider accessibility and integration of mindfulness-based practices into their daily routines. This adaptability aligns with the broader goals of teacher education to support academic success and emotional resilience.

Our findings advocate for educational policymakers globally to recognize the importance of culturally sensitive mindfulness and SEL practices within teacher preparation programs. Such acknowledgment, already explored in Western contexts (Murano et al., 2019; Zimmerman, 2018), underscores the necessity of respecting diverse cultural backgrounds to enhance the effectiveness of educational interventions. The success of the culturally adapted MBSEL program provides a scalable model for fostering teacher resilience, satisfaction, and competence, which can inform global educational reform.

By prioritizing teacher wellbeing, initiatives like MBSEL foster resilience against stress and burnout, contributing to more empathetic and cooperative classroom environments. This, in turn, supports holistic student success, as evidenced by the positive feedback and satisfaction scores reported by participants (Garro et al., 2023). The iterative refinement of MBSEL through research-practice partnerships, as recommended by Coburn and Penuel (2016), further ensures its adaptability and relevance, making it a dynamic and impactful tool for improving educational climates.

### 4.2 Applicability of the MBSEL program in other countries

The Mindfulness-Based Social and Emotional Learning (MBSEL) program, fundamentally rooted in the universal practices of mindfulness and SEL, indeed holds significant promise for global educational systems, extending well beyond the specific cultural context of China. Its core principles—namely enhancing mindfulness, self-compassion, and life satisfaction—are crucial for educators worldwide who are grappling with challenges such as stress and burnout. For its successful international application, the program necessitates thoughtful cultural adaptation, ensuring it aligns seamlessly with local traditions, values, and educational practices. Accordingly, adaptation strategies might include customizing mindfulness exercises to reflect local meditative traditions and tailoring SEL activities to suit the unique social dynamics and cultural narratives of different communities. Importantly, collaboration with local educators and experts emerges as a key to this adaptation process, facilitating the development of a curriculum that resonates with each unique educational setting.

Furthermore, implementing pilot programs across various countries could offer invaluable insights into the program’s flexibility and effectiveness, thereby allowing for continuous refinement. This approach not only underscores the MBSEL program’s potential as a transformative tool for teacher education globally but also fosters environments that support the wellbeing and professional development of educators and students alike. By emphasizing cultural sensitivity and leveraging the universality of its foundational elements, the MBSEL program can thus be a valuable asset in the global pursuit of enhanced educator well-being and more effective, compassionate teaching and learning environments.

### 4.3 Limitations and future directions

Acknowledging our study’s limitations points to opportunities for future research to refine the findings. First, the challenge posed by our small and less diverse sample comprising 95% female participants limits the generalizability across China’s varied pre-service teacher demographics. Future studies should aim for larger, more diverse participant groups with a more balanced gender representation to ensure broader applicability and enhance the study’s relevance across different educational landscapes.

Second, the quasi-experimental design’s lack of random assignment curtails our capacity to assert causality for the benefits observed. Implementing randomized controlled trials (RCTs) in future investigations would solidify the evidence for MBSEL’s effectiveness and provide clearer insights into its impacts.

Third, the short duration of the MBSEL program and immediate post-intervention assessments raise questions about the longevity of its benefits. Longitudinal research is crucial, aiming to monitor the enduring effects of mindfulness and SEL practices on pre-service teachers, particularly regarding teacher retention, job satisfaction, and student outcomes.

Lastly, our reliance on self-reported measures introduces a potential bias, as these measures are subjective and can be influenced by the participants’ current emotional state or social desirability. To overcome this limitation, future studies should aim to include objective measures such as behavioral observations or physiological indicators. Collecting data from multiple sources, such as peer assessments, classroom observations, or even biological markers of stress and wellbeing, would offer a richer and more accurate evaluation of the intervention’s effects. This multi-method approach would reduce the dependence on subjective participant reporting and enhance the validity of the findings.

## 5 Conclusion

In conclusion, our study highlights the effectiveness of the MBSEL program in enhancing the wellbeing of Chinese pre-service teachers by integrating mindfulness practices with CASEL’s SEL framework. This integration improved mindfulness, self-compassion, and life satisfaction, aligning with key SEL competencies such as self-awareness, self-management, and social awareness. The program’s culturally responsive design, incorporating traditional practices like Zhan Zhuang and Ba-Duan-Jin, underscores the importance of tailoring interventions to specific cultural contexts to enhance engagement and impact. These findings not only demonstrate the potential of mindfulness to complement SEL in fostering educator resilience and professional growth but also offer a scalable model for application in diverse educational settings worldwide. Further research is encouraged to adapt and expand the MBSEL curriculum globally, supporting educators’ wellbeing and creating more supportive learning environments for both teachers and students.

## Data Availability

The data supporting the findings of this study have been made publicly available on the Open Science Framework (OSF) repository and can be accessed at https://osf.io/e27zd.

## Appendix

The 16 activities in the mindfulness-based social and emotional learning curriculum

1. Focused Mindful Breathing: Participants concentrate on their breathing, focusing on the sensation of air entering and leaving the body, which promotes relaxation and focus.
2. Mindful Walking: This involves walking slowly and deliberately, paying close attention to the body’s movement and the sensation of each step, fostering mindfulness in motion.
3. Body Scan Meditation: Participants mentally scan their body from head to toe, noticing sensations and learning to experience them without judgment.
4. Zhan Zhuang (Standing Meditation): A traditional Chinese practice where participants maintain a specific standing posture, focusing on bodily sensations and stability to cultivate internal energy.
5. Square Breathing (Box Breathing): This breathing technique involves inhaling, holding the breath, exhaling, and holding again, each for the same number of counts, which helps regulate the breathing pattern and calm the mind.
6. Chinese Ba-Duan-Jin Practice (Eight Pieces of Brocade): A series of eight exercises that combine movement and breathing to enhance physical health and mental clarity.
7. Tai Chi: A gentle form of martial arts, focusing on slow, controlled movements and deep breathing to enhance physical and mental wellbeing.
8. Red-Green Light Game: A playful activity that teaches self-control and awareness by responding to cues such as ‘red’ for stop and ‘green’ for go.
9. Compassion/Loving-Kindness Meditation: Participants cultivate feelings of kindness and compassion towards themselves and others, enhancing emotional resilience and empathy.
10. Empty Chair Practice: A therapeutic exercise where participants speak to an empty chair imagining another person or themselves in a different state, facilitating emotional expression and processing.
11. Mindful Listening and Communication Practice: This involves actively listening without judgment and responding thoughtfully in conversations, enhancing interpersonal relationships.
12. Social Role-Playing: Participants act out various social situations, which helps them develop social skills and better understand social dynamics.
13. Emotions-Observing Meditation: This practice involves observing emotions as they arise, without attachment, learning to understand and regulate emotional responses.
14. Mindful Communication Journal: Participants record their daily communications, reflecting on their interactions to improve mindfulness in how they communicate.
15. Five Senses Treasure Hunt: A sensory awareness activity where participants focus on experiencing their environment through all five senses, which enhances their ability to live in the present moment.
16. Active Witnessing: This involves being fully present and attentive during interactions or events, observing without judgment or reaction.
